# *In silico* simulations of diffusion tensors and tortuosity in cells grown on 3D-printed scaffolds for tissue engineering

**DOI:** 10.1039/d4ra05362a

**Published:** 2024-10-14

**Authors:** Topaz A. A. Cartlidge, Yan Wu, Thomas B. R. Robertson, Orestis L. Katsamenis, Giuseppe Pileio

**Affiliations:** a School of Chemistry, University of Southampton SO17 1BJ UK g.pileio@soton.ac.uk; b μ-VIS X-ray Imaging Centre, University of Southampton SO17 1BJ UK

## Abstract

Tissue engineering is set to revolutionise regenerative medicine, drug discovery, and cancer biology. For this to succeed, improved 3D imaging methods that penetrate non-invasively into the developing tissue is fundamental to guide the design of new and improved 3D supports. In particular, it is very important to characterise the time- and space-heterogeneous pore network that continuously changes as the tissue grows, since delivery of nutrients and removal of waste is key to avoid the development of necrotic tissues. In this paper, we combine high-resolution microfocus Computed Tomography (μCT) imaging and *in silico* simulations to calculate the diffusion tensor of molecules diffusing in the actual pore structure of a tissue grown on 3D-printed plastic scaffolds. We use such tensors to derive information about the changing pore network and derive tortuosity, a key parameter to understand how pore interconnection changes with cell proliferation. Such information can be used to improve the design of 3D-printed supports as well as to validate and improve cell culture protocols.

## Abstract 2

Tissue engineering is set to revolutionise regenerative medicine, drug discovery, and cancer biology.

## Graphical abstract

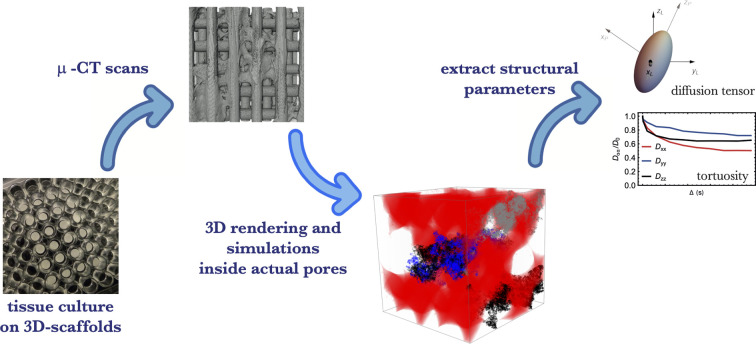

## 1 Introduction

Tissue engineering is a field of science that primarily combines the principles of biology and engineering to develop artificial tissues as functional substitutes for damaged tissues in regenerative medicine applications.^1^ One of its approaches, involving cells cultured on biocompatible matrices, has recently generated a lot of interest within the scientific community. In particular, with the development of novel 3D-printing technologies, a lot of scientific interest and efforts have been focused on growing tissues on 3D-printed plastic supports, also known as cell scaffolds. These are 3D structures printed from biocompatible synthetic polymers (polycaprolactone (PCL), polylactic acid (PLA), and polyglycolic acid (PGA) are very popular) in various shapes, fibre thicknesses, and geometric arrangements.^2^ Such interest is motivated by the huge potential of this approach to revolutionise not only regenerative medicine^3,4^ but also other fields of medicine such as drug discovery^5^ and cancer biology,^6–10^ to cite a few. For example, 3D tumour models grown on 3D-printed plastic scaffolds are under intense scrutiny since they can provide a more realistic *in vitro* representation of a solid tumour by better mimicking the tumour's microenvironment.^11^ Traditional 2D tumour models, where cells are grown on 2D plates with no extracellular matrix environment present, have, in contrast to 3D models, an unnatural proliferation kinetics, altered behaviour, and even an altered reaction to toxicants, so much that they are not really considered appropriate cancer models.^9^ Similarly, 2D cell cultures grown on plastic surfaces, despite ubiquitous in cellular assays used for high-throughput screening, are not regarded anymore as representative of physiological conditions and therefore drug tested on such models are likely to fail at later stages in the clinical trials.^5^

Producing scaffolds with the right characteristics for a given application is, however, still an open challenge. Physiologically relevant models require careful consideration of many concomitant factors^12^ including: the technical design of the 3D support, in terms of fibre size, arrangement, and spacing; the fibre coating biomaterial, required to facilitate cell adherence as well as to recreate the correct extracellular matrix that would support the tissue growth and cell migration; the diffusion of nutrients and removal of cellular waste, essential factors for maintaining the cell culture's viability; the monitoring of the tissue growth inside the millimetre-sized scaffolding, crucial for interrogating the cells in practical applications and in optimising culture protocols in the development phase. Of relevance to what is discussed in this paper, the diffusion of nutrients and waste is not a trivial problem to solve nor to characterise, due to the lack of suitable 3D techniques that provide a direct measurement of molecular diffusion and, more importantly, tortuosity. The issue is clearly complicated by the fact that the 3D scaffolding is a complex porous system which is alive, has millimetric size, and is heterogeneous in space and time, *i.e.*, cell proliferation can be patchy in space but also in time and therefore the porous structure itself, and consequently, the molecular dynamics within, is different in different locations and at different times. As the cells proliferate and infiltrate the pores of the scaffolding, the diffusion of molecules within the pores becomes restricted and the path molecules take while travelling between distant pores becomes more and more tortuous. A characterisation technique suitable to measure diffusion and tortuosity of the growing tissue therefore has to be non-destructive, able to penetrate for millimetres into the sample and, most importantly, able to provide not only structural but also dynamical and functional information. These requisites seem to fit well with the features of magnetic resonance and part of the efforts of our laboratory are concentrated on developing a diffusion tensor imaging technique able to measure diffusion and tortuosity in those systems, which we will report of elsewhere. In this paper, we make use of the abilities of μCT and combine those with customised *in silico* simulations to predict the diffusion tensor as a function of the molecular diffusion time. In literature, there is plenty of algorithms that use random-walk based simulations to derive diffusion and tortuosity in a variety of porous media including rocks, soils, empty scaffolds and tissue systems.^13–16^ The structure of these systems is often idealized through modelling. In here, we apply these well-established techniques to the case of tissues growing on 3D-printed supports with the attention to catch their exact time and space dependent heterogeneity by a 3D rendering of the porous medium obtained through μCT imaging. This approach allows us to extract the apparent diffusivity, principal directions of diffusion, and tortuosity within the actual porous structure of stem cells cultured on 3D scaffolds, fixed at incremental times during cell proliferation. With its ability to reproduce the dynamics within a 3D rendering of the actual porous structure obtained from processed μCT images, we believe our simulations would be of great use to the community, for example for understanding cell–scaffold interactions, informing the discovery of effective cell-instructive biomaterials, improving the design of biocompatible scaffolds, validating cell culture protocols and providing an alternative approach to the many existing studies of diffusion tensors and tortuosity in tissue engineering.^17–19^ Simulated data will also be relevant in the development and validation of magnetic resonance diffusion tensor imaging techniques, such as those under development in our laboratory, which are aimed at extracting this information on these medically relevant structures.^20^ In the following, we will first detail the preparation of our samples which consist of cells grown on 3D-printed scaffolds and fixed at different time points during proliferation. We will then illustrate the characterisation of these samples and the collection of their 3D structure through μCT imaging. Finally, we will present the simulation algorithm and discuss the simulation data therefore obtained.

## 2 Materials and methods

### 2.1 Preparation of porous model systems

#### 2.1.1 Materials

**Fig. 1:**
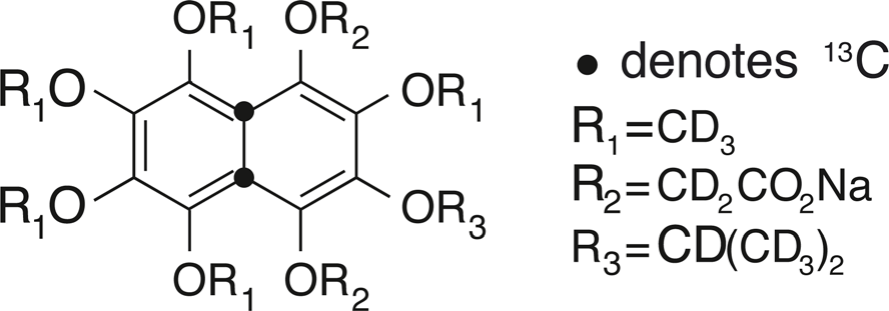
Molecular structure of sodium-2,20-((1,2,3,4,6-pentakis-(methoxy-d_3_)-7-(propan-2-yl-d_7_) naphthalene-5,8-diyl)bis(oxy))diacetate-4a,8a-^13^C_2_.

Two porous model systems were prepared to validate simulation results against experimental measurements. Both systems are made up of randomly packed polyethylene (PE) beads of different diameters. One system (here addressed as PES – PolyEthylene Small) was prepared by randomly packing 500–600 μm diameter beads inside a 10 mm OD NMR sample tube. The other system (here addressed as PEL – PolyEthylene Large) was made by packing 1000–1180 μm diameter beads in a 10 mm OD NMR tube. Note that both these systems were featured in ref. 21, where the experimental data used below are taken from. A replica of these two systems was made by packing beads of the same sizes into custom-made 10 mm OD polycarbonate tubes. These replicas were used for μCT imaging since the strongly X-ray attenuative glass tubes can affect the μCT contrast resolution. For both samples, 3 g of microspheres was added to the tube in a number of small aliquots. At each addition, the sample was gently tapped to improve the packing. For the NMR experiments (data taken from our own previous work^21^), the beads packed in NMR tubes were imbibed with a 0.14 M solution of sodium-2,20-((1,2,3,4,6-pentakis-(methoxy-d3)-7-(propan-2-yl-d7) naphthalene-5,8-diyl)bis(oxy))diacetate-4a,8a-13C_2_ (see Fig. 1) in D_2_O and manually shaken to remove any visible air bubbles. The diffusion tensor of the spy molecule was then measured using the singlet-assisted diffusion-NMR technique described in ref. 21.

#### 2.1.2 μCT imaging

**Fig. 2:**
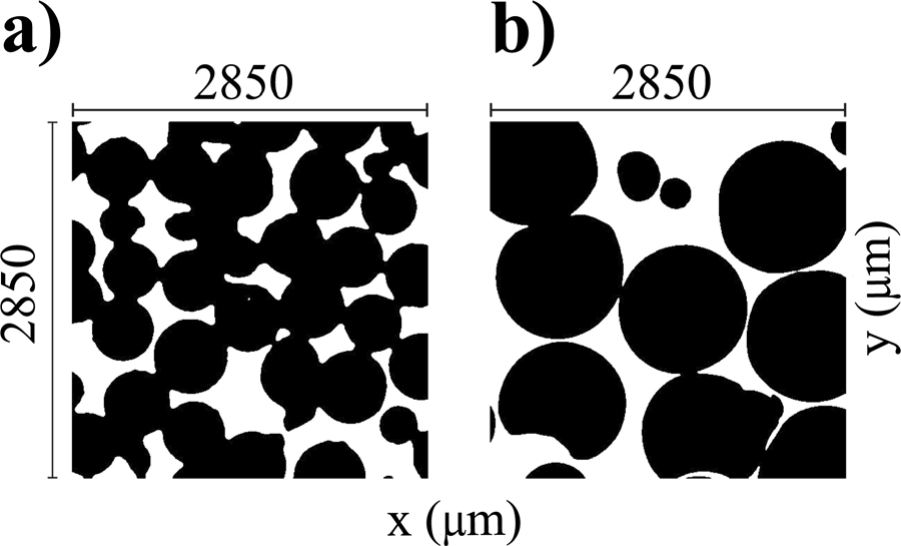
Central *xy*-plane slice of the binarized μCT images taken on the two polyethylene beads packings^21,24^ used in this work to validate simulations: (a) sample PES, bead size 500–600 μm, images at 5.56 μm per pixel; and (b) sample PEL, bead size 1000–1180 μm, images at 5.56 μm per pixel.

To obtain the digitized structure function, *s*(*r*), for these model porous media we used microfocus computed tomography imaging (μCT). CT images of the two model porous media samples described above were collected at the μ-VIS X-ray Imaging Centre (https://muvis.org) using either 160 kV Nikon Med-X prototype or a custom Nikon XTH 225 ST (Nikon X-Tek Systems Ltd) μCT scanner at the centre's 3D X-ray Histology Facility at the University Hospitlal Southampton.^22^ Imaging was conducted at 80 kVp using between 2201 -2501 projections depending on the system used. Scan duration ranged between 1 h 20 min to 1 h 40 min. The sample packings were prepared in custom-made 10 mm poly(carbonate) tubes formed from a cut section of 10 mm outer diameter and 1.5 mm wall-thickness tubing (Clear Plastic Supplies, U.K.). (μCT) images were processed using the ImageJ software.^23^ Raw data comprising the whole sample were cropped to reduce file for simulations (512 × 512 × 512 voxel taken from the centre of the reconstructed volume). These reduced-volume images were filtered and binarized. A slice of each data set is shown in Fig. 2.

Binarization was done using ImageJ's automatic threshold tool with thresholds calculated for each slice and manually checked after binary conversion. A median filter of 3 pixels was applied to the volume before binarization in order to minimise noise and remove artefacts. A further median filter of 2 pixels was applied following binarization. Note, from these μCT images, the non-spherical nature of the beads results in a complex and unpredictable environment that is not easily reproducible by *in silico* produced models of regular spherical beads packing, but rather requires knowledge of the actual pore structure as we do in this work.

### 2.2 Cell culture on 3D-printed scaffolds

An important part of this paper consists of applying the developed simulation framework to space and time heterogeneous porous media of factual importance such as tissues grown on 3D-printed supports.

#### 2.2.1 Materials

**Fig. 3:**
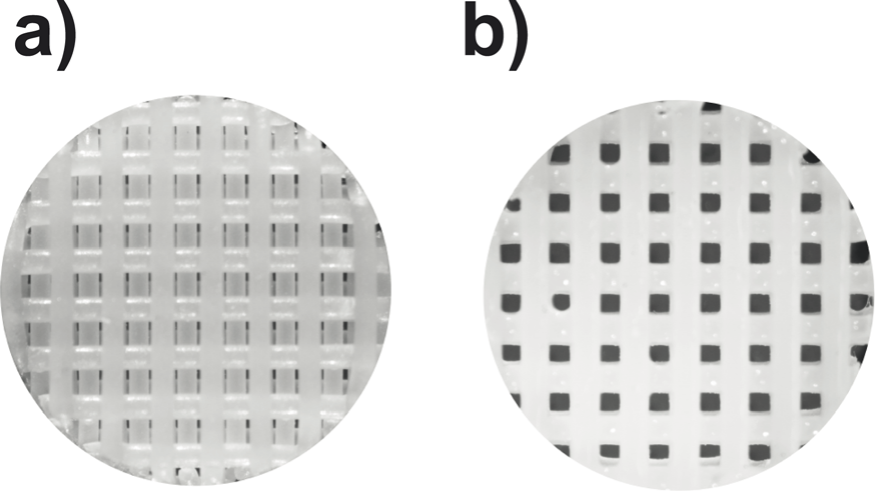
Microscope picture of 3D Insert™ polycaprolactone (PCL) scaffolds with (a) offset and (b) aligned fibres.

The MC3T3-E1 cell line cultivated on the scaffolds as described below, was obtained from the Institute for Developmental Science (IDS) of the Southampton General Hospital (Southampton, UK). Two different sets of 3D Insert™ polycaprolactone (PCL) scaffolds were purchased from 3D Biotek (New Jersey, USA). These cylindrical scaffolds have a diameter of 5 mm and a thickness of 1.5 mm. Their fibres are 300 μm thick and arranged in five layers in a cross-hatch fashion, leaving a 300 μm space between the fibres in each layer (and also among layers). The fibres in each layer are either offset (see Fig. 3A) or aligned (see Fig. 3B) with respect to those two layers above or below. Scaffolds of the aligned type were used for the manual cell culture in 96-well plates whereas those of the offset type were used for the automated culture using a bioreactor.

Trypsin–EDTA solution and Gibco™ heat-inactivated Fetal Bovine Serum (HI FBS) were purchased from Sigma-Aldrich (Missouri, United States). Gibco™ Dulbecco's Modified Eagle Medium (DMEM), Gibco™ Phosphate buffered saline (PBS, pH 7.2), Gibco recovery™ cell culture freezing medium, Fibronectin Bovine Protein (FBP, lyophilized), Penicillin-Streptomycin (P/S, 10 000 U mL^−1^), Nunc™ EasYFlask™ Cell Culture Flasks, Sphera Low-Attachment Surface 96-well plate and pure ethanol were purchased from Fisher Scientific (New Hampshire, United States). Paraformaldehyde fixative solution (PFA, contains 3.7% (w/v) paraformaldehyde and 3% (w/v) sucrose in PBS, pH 7.4) was purchased from Alfa Aesar (Massachusetts, United States). The 3D Perfusion Bioreactor was purchased from 3D Biotek (New Jersey, USA).

#### 2.2.2 Preparation

In order to improve cell adherence onto the plastic scaffolds, we treated all 3D-printed PCL scaffolds with Bovine Fibronectin Protein (BFP). The coating process consisted of the following steps: (i) lyophilized BFP powder was dissolved in PBS at pH = 7.2 in a water bath at 37 °C for 30 min and to a concentration of 100 μg mL^−1^; (ii) scaffolds were fully dipped into the solution for 10 times before they were air-dried in a class II sterile Laminar Flow Hood for 2 hours; (iii) coated scaffolds were stored at 4 °C in a closed Low-Attachment Surface 96-well plate until further use.

#### 2.2.3 Seeding

Frozen MC3T3-E1 cells were thawed in a 37 °C water bath (Clifton NE1-8, Nisbets, Bristol, United Kingdom) for around 2 min before the cell suspension solution was transferred into a sterile Eppendorf tube. The suspension was centrifuged at 300*g* for 2 minutes and the pellet was later suspended in cell culture media (DMEM supplemented with 10% FBS and 1% P/S) to replace the frozen media. The cells were later transferred into cell culture flasks and continually cultured in the incubator at 37 °C and 5% CO_2_. The cell culture medium was replaced every two days to ensure fresh nutrients and waste removal during the cells' growth. In order to prevent overpopulation, for the purpose of reseeding, a trypsin–EDTA solution was used to detach the cells, once the cells had reached 70% confluence. MC3T3-E1 recovered and duplicated within one week of thawing.

#### 2.2.4 Cell culture

Two sets of cultivated scaffolds were produced. For set M (M stands for cultivated Manually), the cells were seeded and cultivated manually in 5 different PCL scaffolds following this procedure: cells were counted with a hemocytometer (Paul Marienfeld GmbH & Co. KG, Germany) and a cell suspension was prepared with a density of 80 000 cells in 20 μL. 20 μL of the cell suspension was then loaded onto every PCL scaffold placed at the bottom of a Low-Attachment Surface 96-well plate. 3 hours after seeding, 200 μL of cell culture medium was added to each well. Seeded scaffolds were kept in the closed cell culture plate and cultured in the incubator until fixation. The cell culture medium was refreshed every 2–3 days. All scaffolds were seeded on the same day but fixed on different days as explained below. For set B (B stands for cultivated in Bioreactor), the cells were seeded manually on 5 different PCL scaffolds but cultivated in an automated bioreactor according to the following procedure: cells were counted with a hemocytometer (Paul Marienfeld GmbH & Co. KG, Germany) and a cell suspension was prepared with a density of 80 000 cells per 20 μL. 20 μL of the cell suspension was then loaded onto each PCL scaffold while placed at the bottom of a Low-Attachment Surface 96-well plate. After 3 hours, the scaffolds were loaded into the sample chambers of the bioreactor (piled on top of each other with an o-ring spacer in between any two scaffolds) and automatically fed with culture medium using a perfusion setting of 1.2 rpm (corresponding to 0.26 ml min^−1^ in our setup) for the first 24 hours, then increased to 6 rpm (corresponding to 1.3 ml min^−1^ in our setup) for the rest of the time. All scaffolds were seeded on the same day and in the same bioreactor chamber but fixed on different days as explained below.

#### 2.2.5 Fixation

At the time of fixation (at days 4, 7, 11, 14 and 17 after seeding), MC3T3-E1 cultivated scaffolds were transferred into a 24-well plate and soaked in paraformaldehyde fixative solution for 15 minutes before being washed three times using 70% ethanol water solution. All fixed samples were stored in a 70% ethanol water solution at room temperature until further use. Table 1 summarises all samples made.

**Table 1:** Naming of cultivated scaffolds used in this work

Name	Cultivation method	Culture time (days)				
		4	7	11	14	17
Set M^a^	Manual	M4	M7	M11	M14	M17
Set B^b^	Bioreactor	B4	B7	B11	B14	B17

a Scaffold type: aligned fibres.

b Scaffold type: offset fibres.

#### 2.2.6 Microscopy

**Fig. 4:**
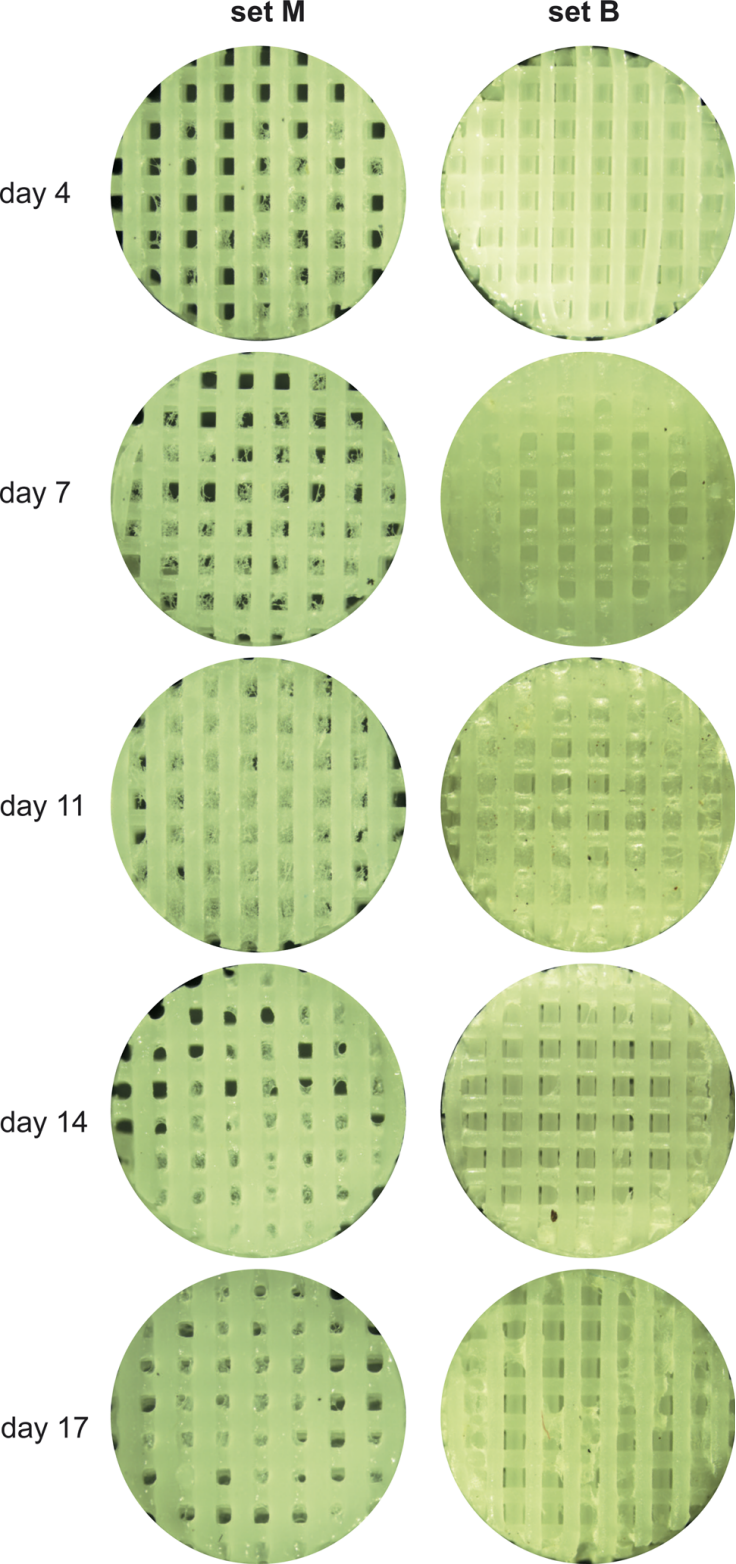
Microscope images of cultivated PCL scaffolds after fixation; set M: cultivated manually in 96-well plates; set B: cultivated in the bioreactor.

All cultivated scaffolds were checked under the microscope after fixation and prior to μCT imaging (see below). For this we have used a Nikon SMZ1000 stereo microscope (Tokyo, Japan) coupled to a Fujitsu DS-L4 tablet-style control unit (Tokyo, Japan) for capturing pictures. All scaffolds were taken out of the storage solution and semi-dried with tissue wipers. X1.5 zoom was used in all pictures (see Fig. 4).

#### 2.2.7 μCT scans

For micro-imaging computed tomography scans, the cultivated scaffolds were stacked in a polycarbonate tube with an internal diameter of 5.2 mm. Each scaffold was separated by a polycarbonate spacing disk cut using a LS3040 CO2 laser cutter (HPC Laser, United Kingdom). The stacks of cultivated scaffolds were scanned using a Nikon XT μCT system using an 80 kVp acceleration voltage, 86 μA current, 6.88 W power and an exposure time of 708 ms. Prior to processing, scans had a voxel size of 3.05 μm in all three dimensions.

To prepare the binarized structure function *s*(*r*) used in the simulation discussed below the reconstructed volumes for each scaffold were converted from 32 bit to 8 bit and then imported into ImageJ for further processing. The data processing ultimately produced a down-scaled binary representation of the original structure and was done following these steps: (i) each image is independently rotated to align the scaffold fibres along the *x* and *y* direction on the *xy*-plane; (ii) a 1024 × 1024 x n volume centred on the scaffold's centre was cut (since the scaffold measures 5 mm OD × 1.5 mm height, the number of orthogonal slices along the *z* direction, *n*, was of the order of 490, with small differences between the scaffolds due to manufacturing variations); (iii) the size of the image was reduced to 512 × 512 × *n*/2 making use of a constrained aspect ratio and averaged bilinear interpolation (this has the effect of doubling the voxel size and therefore halving the initial resolution to 6.1 μm in all three dimensions); (iv) a median filter with a 2 pixels radius was applied; (v) the image was binarised with thresholds calculated for each image separately.

**Fig. 5:**
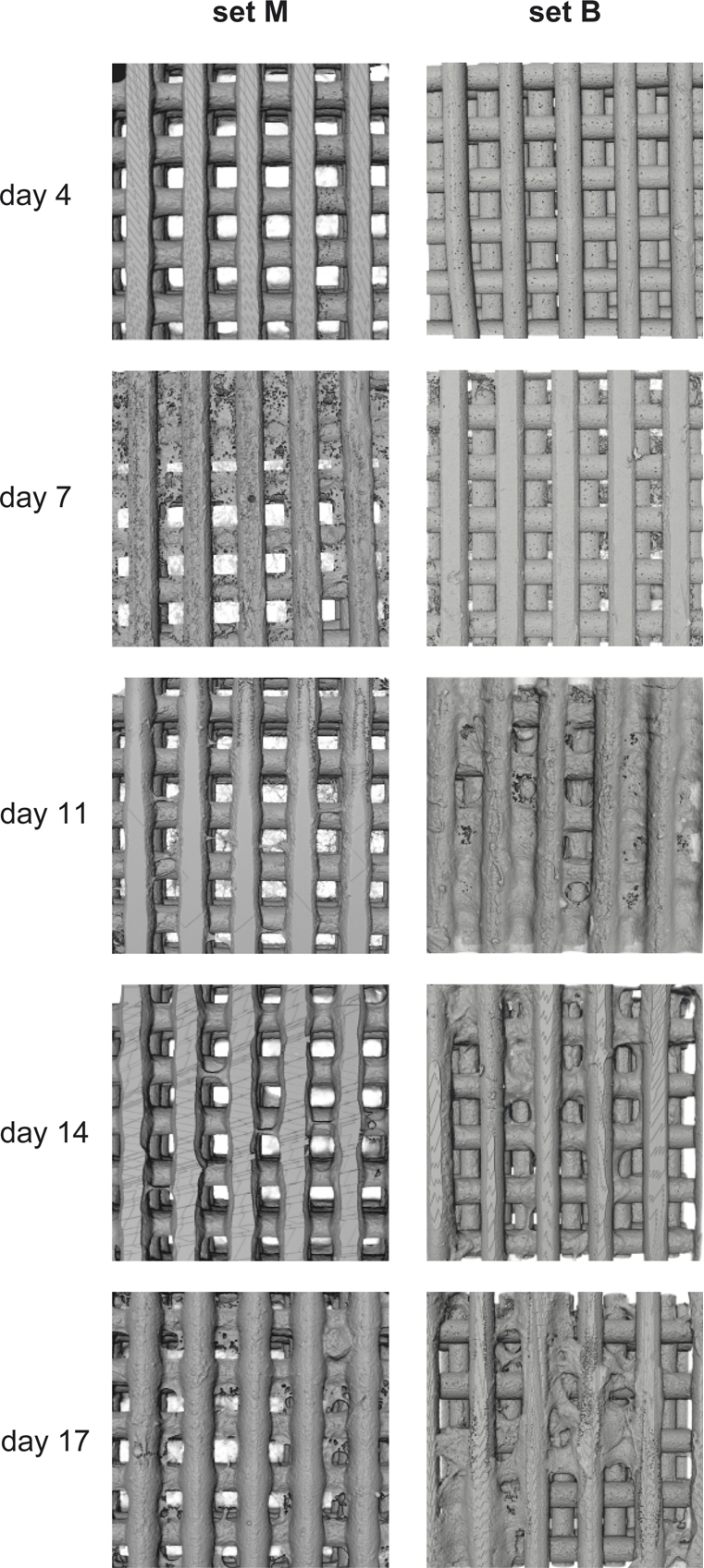
Top-down view in 16 bit grayscale of the reconstructed volume for each scaffold in sets M and B.

Fig. 5 shows a top-down view in 16 bit grayscale of the reconstructed volume for each scaffold in sets M and B (rendering prepared using Avizo 3D 2021.2 software).

## 3 Numerical simulations

### 3.1 Simulation methods

Molecular diffusion through the pores of the actual samples under investigation has been simulated using a random walk algorithm where molecules are allowed to undergo Brownian diffusion within the pores network of the structure, this latter obtained from the digitization of 3D μCT scans. The procedure consists of the following steps:

#### 3.1.1 Digital rendering of the porous structure

A microcomputed tomography 3D image of the porous medium is acquired and binarized to obtain the structure–function, *s*(*r*). This function contains information about the structural nature of the porous structure at each position *r*; namely, *s*(*r*) = 1 if *r* falls within the solid matrix and *s*(*r*) = 0 if *r* falls within a pore. The structure–function is discrete and has the same resolution as the CT scan, it is therefore linearly interpolated to create a finer resolution. In this process, *s*(*r*) can assume any value between 0 and 1 and we will then consider a point to belong to the solid matrix if *s*(*r*) ≥ 0.5 and to be long to a void if *s*(*r*) < 0.5.

#### 3.1.2 Simulation parameters

The number of molecules is set to *N*_m_; the diffusion time *Δ* is chosen and varied across different simulations. The isotropic diffusion coefficient is set to *D*_0_. The number of steps, *N*_s_, is set to 5000 and the duration of each step, *t*_s_, is calculated as *t*_s_ = *Δ*/*N*_s_. The number of steps is carefully chosen so that the average molecular displacement at each step, *i.e.*, 
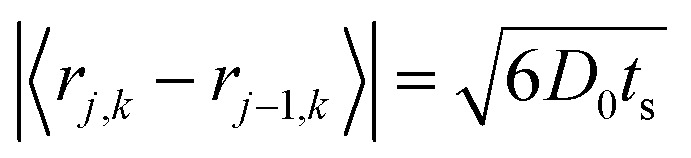
, is much less than the average pore size and the molecule can explore each pore accurately.

#### 3.1.3 Initial configuration

The *N*_m_ molecules are first randomly positioned inside the pores of the structure of interest within a central cube. The central volume of 212^3^ pixels (of the total 512^3^ lattice points) is selected such that the average particle will not encounter the structure boundary within its walk. A vector containing all starting positions is prepared as *R*_i_ = {{*x*_1,0_,*y*_1,0_,*z*_1,0_},{*x*_1,1_,*y*_1,1_,*z*_1,1_},…,{*x*_1,*N*_m__,*y*_1,*N*_m__,*z*_1,*N*_m__}}.

#### 3.1.4 Random steps

For each molecule, *k*, *N*_s_ random steps are taken. At the *j*-th step, the position of the *k*-th molecule is given by *r*_*j*,*k*_ = {*x*_*j*−1,*k*_ + *δx*_*j*,*k*_, *y*_*j*−1,*k*_ + *δy*_*j*,*k*_, *z*_*j*−1,*k*_ + *δz*_*j*,*k*_} with the step increments *δx*_*j*,*k*_, *δy*_*j*,*k*_ and *δz*_*j*,*k*_ chosen randomly from a Gaussian distribution of zero mean and standard deviation 
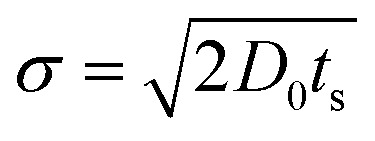
. The position *r*_*j*,*k*_ is checked such that if *s*(*r*_*j*,*k*_) < 0.5 (hence the molecule is within a pore) then the molecular position is updated; conversely, if *s*(*r*_*j*,*k*_) ≥ 0.5, then a new random step is attempted and checked with the same criteria. At the end of the walk a vector containing all final position for each molecule is prepared as *R*_f_ = {{*x*_*N*_s_,0_,*y*_*N*_s_,0_,*z*_*N*_s_,0_},{*x*_*N*_s_,1_,*y*_*N*_s_,1_,*z*_*N*_s_,1_},…,{*x*_*N*_s_,*N*_m__,*y*_*N*_s_,*N*_m__,*z*_*N*_s_,*N*_m__}.

#### 3.1.5 Average square travel distance

The square of the average distance travelled by molecules along 6 different directions is calculated as:1
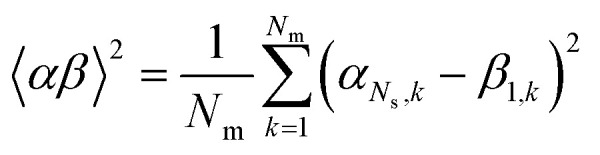
where *α* and *β* indicated any two directions (*α*, *β* = *x*, *y*, *z*).

#### 3.1.6 Diffusion tensor

The diffusion tensor is a rank 2 symmetric matrix with six independent components:2
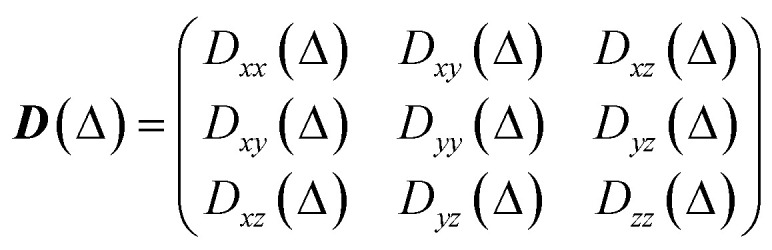
with *D*_*xy*_(*Δ*) = *D*_*yx*_(*Δ*), *D*_*xz*_(*Δ*) = *D*_*zx*_(*Δ*) and *D*_*yz*_(*Δ*) = *D*_*zy*_(*Δ*). In here, we have imposed this symmetry by calculating only six independent components of the diffusion tensor, obtained from the average square travel distance as:3
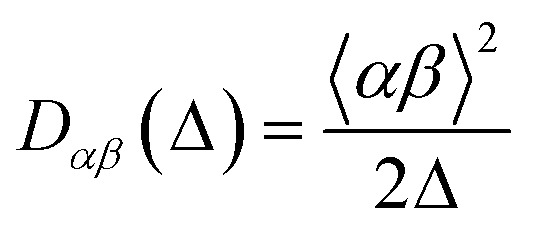


The resulting diffusion tensor is therefore expressed in the laboratory frame. This is then diagonalised to obtain its eigenvectors (*x*′, *y*′, *z*′), corresponding to the three principal directions of diffusion, and its eigenvalues (labelled such that 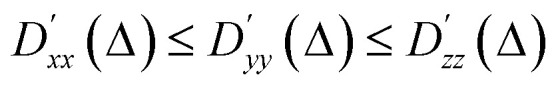), corresponding to the diffusivity along those three principal directions. The diagonal tensor is:4
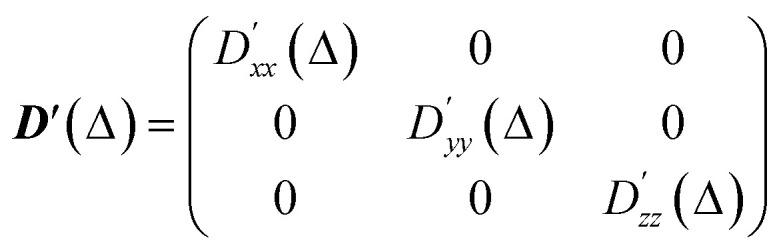


The simulated apparent diffusion coefficient *D*_a_(*Δ*) is obtained as the trace of the tensor:5
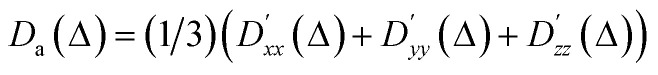


For visualisation, the diffusion tensor is represented by an ellipsoid oriented along the principal direction of diffusion (*x*′, *y*′, *z*′) with semiaxes lengths set to:6
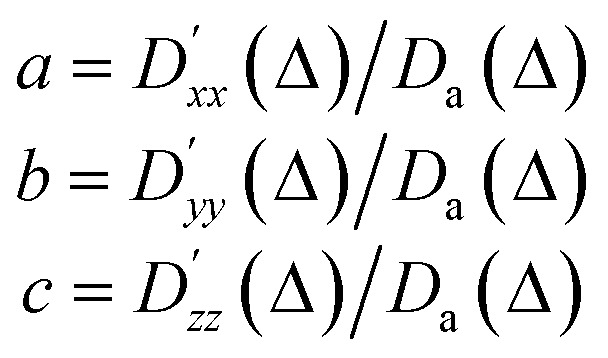


#### 3.1.7 Tortuosity

The tortuosity of the structure, usually indicated by *α* but here indicated by *τ* to avoid confusion (we have used *α* to indicate a generic direction), is extrapolated as the asymptote of the plot of *D*_a_(*Δ*)/*D*_0_*versus Δ*. There are many ways to define tortuosity and relate it to the restricted diffusion coefficient in porous media.^25^ In all cases, though, tortuosity is inversely related to the ratio of restricted *versus* unrestricted diffusion coefficient (*D*_a_(*Δ*)/*D*_0_), and can, in some instances, include porosity^26^ and constrictivity^27^ terms. Here, we adopt the relationship most commonly used in analysis of NMR diffusion data (adapted from ref. 28):7
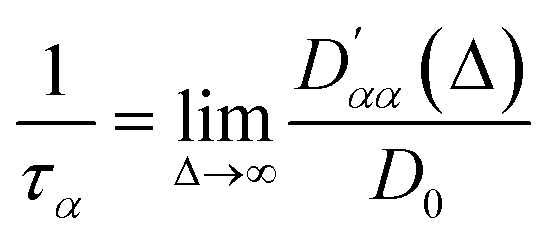


The simulation parameters for a typical simulation (in the case of *Δ* = 60 s) are summarised in Table 2.

**Table 2:** Simulation parameters used in this paper

Parameter	Value
*N*_m_	20 000
*D*_0_	2.2 × 10^−10^ m^2^ s^−1^
*N*_s_ (for *Δ* = 60 s)	5000
*τ*_s_ (for *Δ* = 60 s)	12 ms
|〈*r*_*j*,*k*_ − *r*_*j*−1,*k*_〉| (for *Δ* = 60 s)	4.0 μm
|〈*r*_*N*_s_,*k*_ − *r*_1,*k*_〉| (for *Δ* = 60 s)	281.4 μm

### 3.2 Validations of simulation algorithm

Numerical simulations were written in both Julia and Wolfram Mathematica language. To validate the simulation framework, a number of simulations were run for a few different porous structures (created *in silico* or experimentally produced) and their results, in terms of the diffusion tensor, the diffusion ellipsoid, fractional anisotropy, and the tortuosity were compared with what was expected or what was experimentally measured. The chosen structures ranged from an empty spherical container to a model porous system for which we have experimental data. For each structure, the boundary of the structure–function was treated as an external solid wall, meaning any diffusing molecule encountering the walls would be reflected back, so to prevent large jumps in particle position. Tortuosity was calculated by varying the diffusion time (*Δ*) from 0 to 240 seconds and determining the ratio of *D*_a_(*Δ*)/*D*_0_ for each case. The asymptotic value of the plot of *D*_a_(*Δ*)/*D*_0_*versus Δ* is proportional to the tortuosity of the system. The degree of anisotropy with respect to diffusion within the structure was quantified in terms of the fractional anisotropy (FA), defined as:^29^8
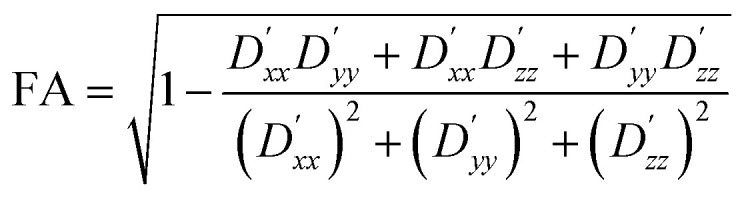


FA ranges from zero, indicating isotropic unrestricted diffusion, to one, indicating completely anisotropic diffusion.

#### 3.2.1 Empty sphere

**Fig. 6:**
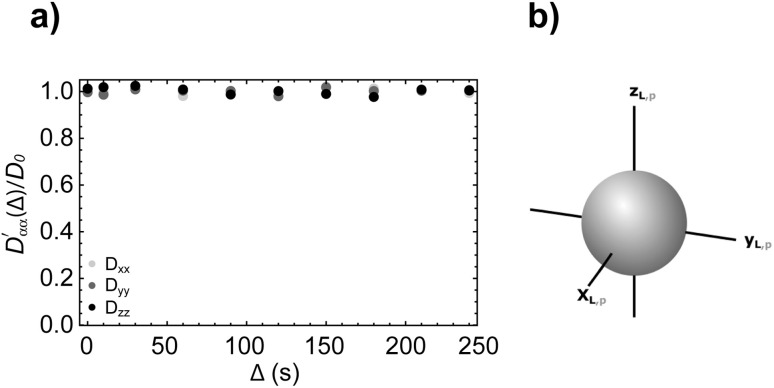
(a) Simulated diffusion coefficients along the three principal axes (normalised to *D*_0_) plotted against the diffusion time *Δ* and calculated with our simulation algorithm for molecules diffusing inside an empty sphere (simulation parameters in Tables 2 and 3). (b) The resulting diffusion ellipsoid built using eqn (6).

A first simulation was run using an empty sphere as the space in which molecules can undergo Brownian diffusion. This structure was chosen in order to test the capability of the algorithm to capture isotropic unrestricted diffusion where no boundaries are present. In such a structure, it is expected that diffusion is identical along every direction originating from the centre of the sphere. Additionally, within the volume of the sphere, excluding the surface layer in contact with the walls, diffusion should not be restricted. The spherical structure was implemented using a 512^3^ binary array containing a centre sphere of radius 2.5 mm where each pixel within this volume corresponds to a value of *s*(*r*) = 0, indicating free pore space. All remaining pixels were set to a value of *s*(*r*) = 1 which creates a solid wall for diffusing molecules. The structure function was interpolated to increase the fineness of mesh within which the molecules diffuse. To prevent any effect from the boundary, the starting positions for all the molecules were set to fall within a cubic region with a width of ∼2 mm centred at the very centre of the sphere. Due to the diffusion coefficient of the molecules and the distance to the wall, it was not anticipated that any molecules would encounter the wall in the diffusion times set in this simulation. The parameters used for this simulation are given in Tables 2 and 3, where the average distance travelled by molecules during the longest walk (*Δ* = 240 s) is also reported. For a range of diffusion times from 0 to 240 s diffusion was calculated along 6 directions: *xx*, *yy*, *zz*, *xy*, *xz* and *yz*. The diffusivities along these directions were used to build the diffusion tensor and this was diagonalized to find the principal directions of diffusion and the diffusion coefficients along those principal directions. The circles in Fig. 6 represent the simulated values of 
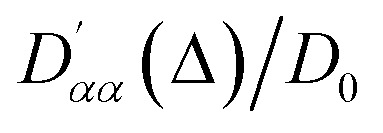
 (with *α* = *x*, *y*, *z*) plotted *versus* the diffusion time *Δ*.

**Table 3:** Simulation parameters for spherical structure

Parameter	Size (px)	Size (mm)
Resolution	1	9.8 × 10^−3^
Sphere radius	256	2.5
Initial volume containing molecules	212^3^	2.07^3^
Av. distance travelled in 240 s	57.6	0.56
Actual distance travelled in 240 s	53.1	0.52

The figure shows that the diffusion coefficients along the three principal axes are essentially identical to one another at any value of *Δ*, meaning that, as expected, the diffusion tensor is isotropic with three identical eigenvalues. Furthermore, since none of the principal diffusion coefficients varies with *Δ* we can conclude that the diffusion is, again as expected, unrestricted. The simulated value of the diffusion tensor in its principal frame and for *Δ* = 240 s is:
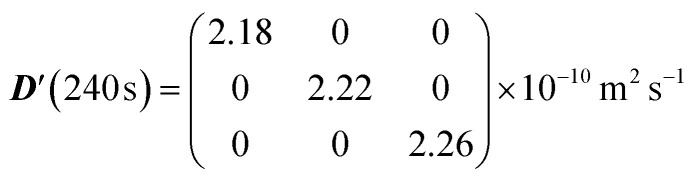


The trace of this tensor (divided by 3) returns an isotropic diffusion coefficient of 2.22 × 10^−10^ m^2^ s^−1^, which matches the diffusion coefficient used for the simulation (see Table 2); the very small deviation can be attributed to a few molecules potentially interacting with the sphere wall, or systematic errors arising due to the limited sample size and number of molecules used in the simulation. Therefore, it can be concluded that the simulation is capable of capturing isotropic, unrestricted diffusion.

#### 3.2.2 Long, thin and tilted cylinder

**Fig. 7:**
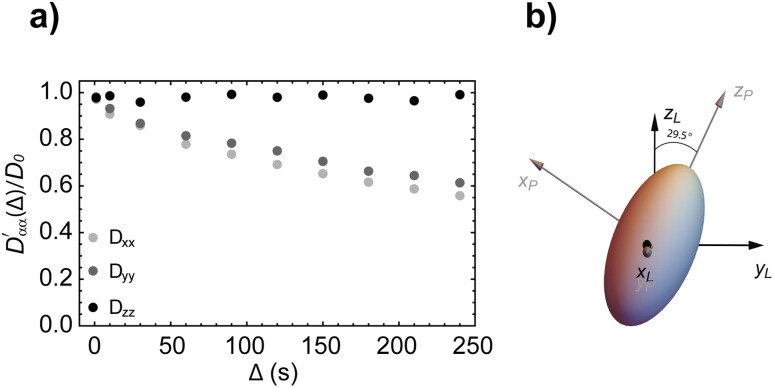
(a) Simulated diffusion coefficients along the three principal axes (normalised to *D*_0_) plotted against the diffusion time *Δ* and calculated with our simulation algorithm for molecules diffusing inside a long and thin cylinder tilted by 30° about the *x*-axis (simulation parameters in Tables 2 and 4). (b) The resulting diffusion ellipsoid built using eqn (6).

In a second validation step, the simulation algorithm was run with molecules let to diffuse within a cylinder of 10 mm in length and 0.5 mm in radius, tilted by 30° about the *x*-axis. As above, the volume within the cylinder corresponds to pixels with an assigned value of *s*(*r*) = 0, thus indicating a void pore. In analogy to the spherical structure above, we used interpolation to allow for a more accurate definition of the space. Such a cylindrical structure was chosen to test the capability of the simulation algorithm to determine the correct anisotropy and orientation of the diffusion tensor. Simulations were run using the parameters given in Tables 2 and 4. As in the previous case, molecules were all initially placed within a small cubic volume at the centre of the cylinder but this time they were given enough diffusion time to hit the side walls of the cylinder but not its top and bottom circular faces. Therefore, it was expected that diffusion along *z* (the long axis of the cylinder) would be essentially free whilst diffusion along the *x* and *y* directions would be significantly restricted. The results of these simulations are reported in Fig. 7, where the simulated values of 
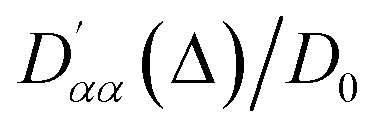
 (with *α* = *x*, *y*, *z*) are plotted *versus* the diffusion time *Δ* (a) and the resulting diffusion ellipsoid is displayed (b). As expected, the shape of the diffusion ellipsoid, almost with perfect cylindrical symmetry and prolate along the *z*-axis, reports an unrestricted diffusion along the *z*-axis and a significant restriction in both the *x* and *y* axes. The plot in panel a of Fig. 7 also shows how the diffusion coefficient along the *z*-axis very rapidly diverges (and remains bigger) from the value of the diffusion coefficient in the other two directions. The simulated value of the diffusion tensor in its principal frame and for *Δ* = 240 s is:
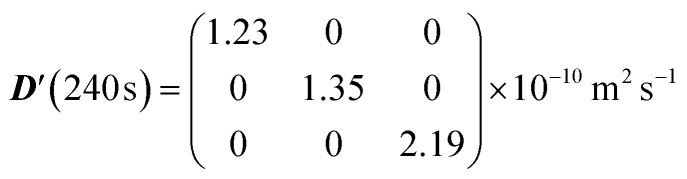
which shows nearly cylindrical symmetry in *x* and *y* and a diffusion coefficient along the *z*-axis which is almost identical to the unrestricted diffusion coefficient used in the simulation (see Table 2). Furthermore, the angle between the principal diffusion axis, *Z*_P_, and the laboratory frame *z*-axis, *Z*_L_, is calculated as 29.5° which is very close to the nominal value of 30° used in the simulation, the small deviation being interpreted as a numerical error due to a finite number of molecules being averaged.

**Table 4:** Simulation parameters for 30° tilted cylinder

Parameter	Size (px)	Size (mm)
Resolution	1	16.9 × 10^−3^
Initial volume containing molecules	212^3^	3.6^3^
Cylinder radius	29	0.5
Cylinder length	588	10
Av. distance travelled in 240 s	33.3	0.56
Actual distance travelled in 240 s	25.7	0.44

#### 3.2.3 Model porous systems

In a final validation step, we aimed to compare simulation predictions with experimental data gathered on two model porous systems made by randomly packed beads of two different ranges of diameters (see Material and methods). Simulations on those two model systems were run using the parameters reported in Tables 2 and 5 and the algorithm described above.

**Table 5:** Simulation parameters for the two model porous systems

Parameter	Size (px)	Size (mm)
Resolution	1	5.5 × 10^−3^
Initial volume containing molecules	212^3^	1.18^3^
Av. bead width (PES)	98.8	0.55
Av. bead width (PEL)	189.4	1.05
Av. distance travelled in 240 s	101.3	0.56
Actual distance travelled in 240 s (PES)	68.3	0.38
Actual distance travelled in 240 s (PEL)	76.7	0.43

**Fig. 8:**
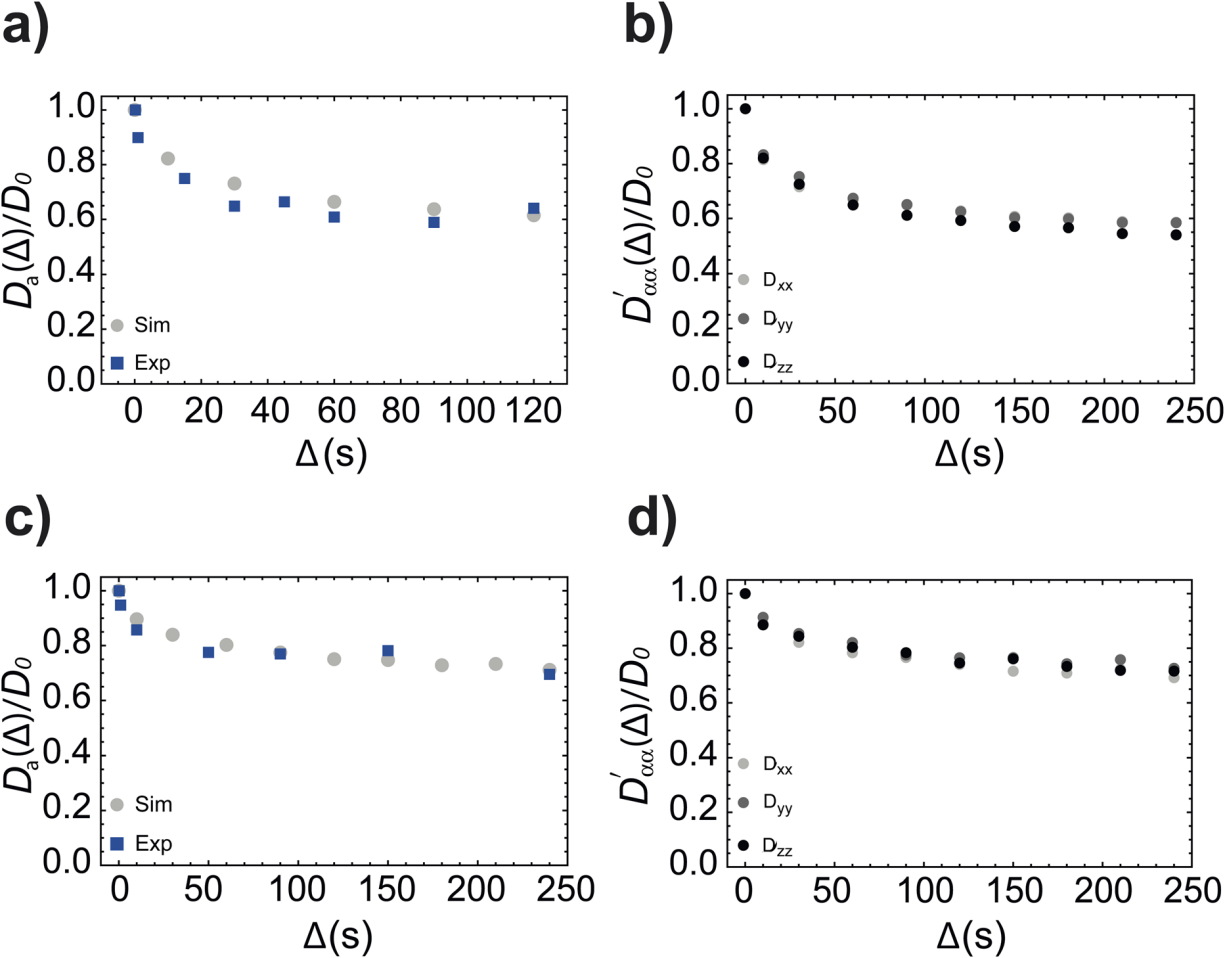
Simulated value of the trace of the diffusion tensor in PES (a) and PEL (c) (grey circles, normalised to *D*_0_) plotted against the diffusion time *Δ* and compared to experimental values of the same quantity (blue squares) measured by NMR in ref. 21. Simulated diffusion coefficients (normalised to *D*_0_) along the three principal axes plotted against the diffusion time and calculated with our simulation algorithm for molecules diffusing inside PES (b) and PEL (d) beads. Simulation parameters are in Tables 2 and 5.

Fig. 8a and c compare the simulated value of the trace of the diffusion tensor in PES and PEL (grey circles) *versus* the experimental value measured with singlet-assisted diffusion NMR techniques in ref. 21 and 24 as a function of the diffusion time *Δ*. The three simulated principal components of the diffusion tensor (normalised to *D*_0_) are plotted in Fig. 8b and d for comparison. All simulated results match very closely with the available experimental data and this highlights the ability of our simulation framework to properly catch and quantify molecular diffusion (and related quantities) within a porous structure of somewhat arbitrary complexity.

Furthermore, since the diffusion coefficient is essentially identical along the three principal directions (for both PES and PEL) we can conclude that the is very little anisotropy in those model systems. This is expected since although the systems offer a non-spherical array of varying sized beads, the packing is sufficiently random that no preferred diffusion direction is available to molecules.

Table 6 shows the simulated and experimental values of tortuosity and the simulated fractional anisotropy for PES and PEL samples as resulting from our simulations and experiments (the experimental value of tortuosity is assumed to be the asymptotic value of the experimental points in Fig. 8a and c). The simulated values are sufficiently close to the experimental values. As expected, the system with the smaller beads, and therefore smaller pores (*i.e.*, PES), shows a larger tortuosity value with respect to the one with larger beads, and therefore larger pores (*i.e.*, PEL).

**Table 6:** Tortuosity and fractional anisotropy of PES and PEL samples resulting from experimental and simulated data

Sample	Tortuosity		FA
	Exp	Sim	
PES	0.61^a^	0.56	0.058
PEL	0.69^a^	0.70	0.025

a Experimental data from ref. 21.

## 4 Diffusion and tortuosity in tissues grown on 3D scaffolds

**Fig. 9:**
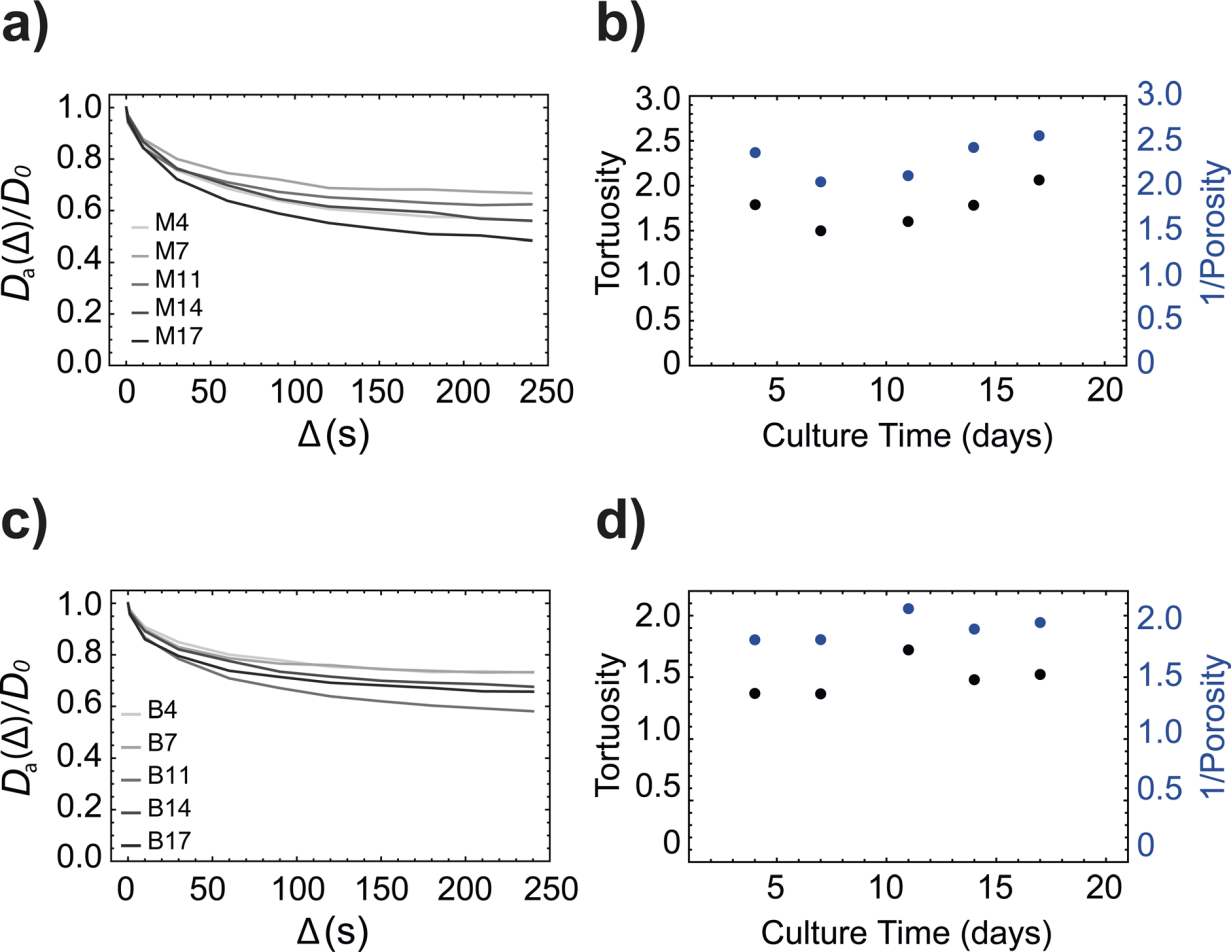
Simulated values of the trace of the diffusion tensor (solid line, normalised to *D*_0_) plotted against the diffusion time *Δ* for samples (a) M4–M17 and (c) B4–B17. The tortuosity (black circles, *τ* in eqn (7), calculated using the trace values of the simulated diffusion tensor) plotted against the culture time and compared to the inverse of porosity (blue circles) for samples (b) M4–M17 and (d) B4–B17.

Given the successful validation of the simulation algorithm discussed above, we applied it to simulate tortuosity in real porous samples made by cells grown on 3D-printed plastic scaffolds (see Material and methods). These samples are challenging but fascinating at the same time since these structures are heterogeneous in both space (as cells grow more or less randomly in the 3*D* space) and time (as they proliferate differently in time). To try to highlight the utility of our simulation framework for porous media of such a kind, we prepared two different sets of cell cultures using two different scaffold types (offset or aligned) but also two different culture techniques (manual, in wells or automatic, *via* a bioreactor). There has been no interest at this stage, and therefore no efforts were made, to refine the culture protocol to obtain reproducible cultures or a consistent set of fixed tissues, simply because this was not the purpose of this paper. Rather, we produced a number of heterogeneous porous media and used our algorithm to extract some structural information from the simulated diffusion tensor. As discussed above, the starting point of our simulations consists in the digitised image of the actual sample which makes our procedure able to cope with media of arbitrary complexity such as those below. The μCT images of all samples used here are reported in Fig. 5. Starting from the set of tissues cultivated manually (samples M4–M17 in Table 1), Fig. 9a shows the results of our simulations by plotting the value of the trace of the simulated diffusion tensor (normalised to *D*_0_) *versus* the diffusion time using the simulation parameters in Table 2. As above, the molecules are originally randomly distributed within a cubic volume at the centre of the structure and spanning from pixel 150 to pixel 362. These results show that at small diffusion times, all the structures seem to have similar tortuosity as if the cells have not proliferated much during the culture time. However, this is a false conclusion since tortuosity requires long diffusion times to be accurate. When the diffusion time is sufficiently long, these curves reach an asymptotic value that is the inverse of tortuosity. In the present set of samples, this asymptotic value becomes lower as the culture time increases, which essentially means that molecular diffusion within these scaffolds becomes more and more tortuous as the cells proliferate. The scaffold fixed at day 4 seems to be an outlier since it reaches the same asymptotic value reached by the one fixed at day 11. However, seeding and successive proliferation of cells depends on many factors and it is expected that some cells have adhered better to a scaffold rather than another one. In a tentative to rationalise this, we have hypothesised that tortuosity is inversely proportional to porosity (*ϕ*) through some unknown equation. In fact, many of these equations exist in literature for different kinds of porous media,^30^ the possibly most known one being 1/*τ* = (1/*ϕ*)^−1/2^, used for randomly packed spheres.

Fig. 9b shows a double-scale plot displaying both tortuosity (on the left-hand scale) and inverse porosity (on the right-hand scale) plotted against culture time. The figure shows a clear inverse correlation between the two quantities. It also shows how the high value of tortuosity found for the culture fixed at day 4 is not unexpected but rather consistent with the cell coverage reached on that particular scaffold at fixation time, which is comparable to that observed in sample M14, possibly due to a better seeding or adherence of cells on that particular scaffold.

An identical set of simulations was run for the five scaffolds cultivated using the bioreactor (samples B4–B17 in Table 1). As in the previous case, the simulated value of the trace of the diffusion tensor (solid line, normalised to *D*_0_) plotted against the diffusion time *Δ* in Fig. 9c, shows that the asymptotic value related to tortuosity can be reached at long diffusion times and that this value becomes lower as the culture time is incremented, with the only anomaly of sample B11 for which the asymptotic value is found to be the lowest of the whole set, despite it was cultivated for less time than B14 and B17 samples. Once again, this anomaly can be easily correlated to the particularly good seeding, adherence and/or proliferation of the cells in this particular scaffold. Indeed, even in this case, the value of tortuosity correlates well with the inverse of porosity (see Fig. 9d) and sample B11 displays a lower porosity, which justifies the reasoning above.

More generally, the absolute values of tortuosity calculated for the M4–M17 set are all higher than those calculated for the B4–B17 set (compare the black circles in Fig. 9b with those in Fig. 9d). This means that the manual protocol has, in this instance, performed better than the bioreactor. This should not be generalised by any means since, as previously remarked, there was no effort to optimise protocols in this work. For example, we became aware at the reviewing stage that the relatively high perfusion settings of 1.3 ml min^−1^ used may have caused cell death due to excessive shear stress.^31,32^

**Fig. 10:**
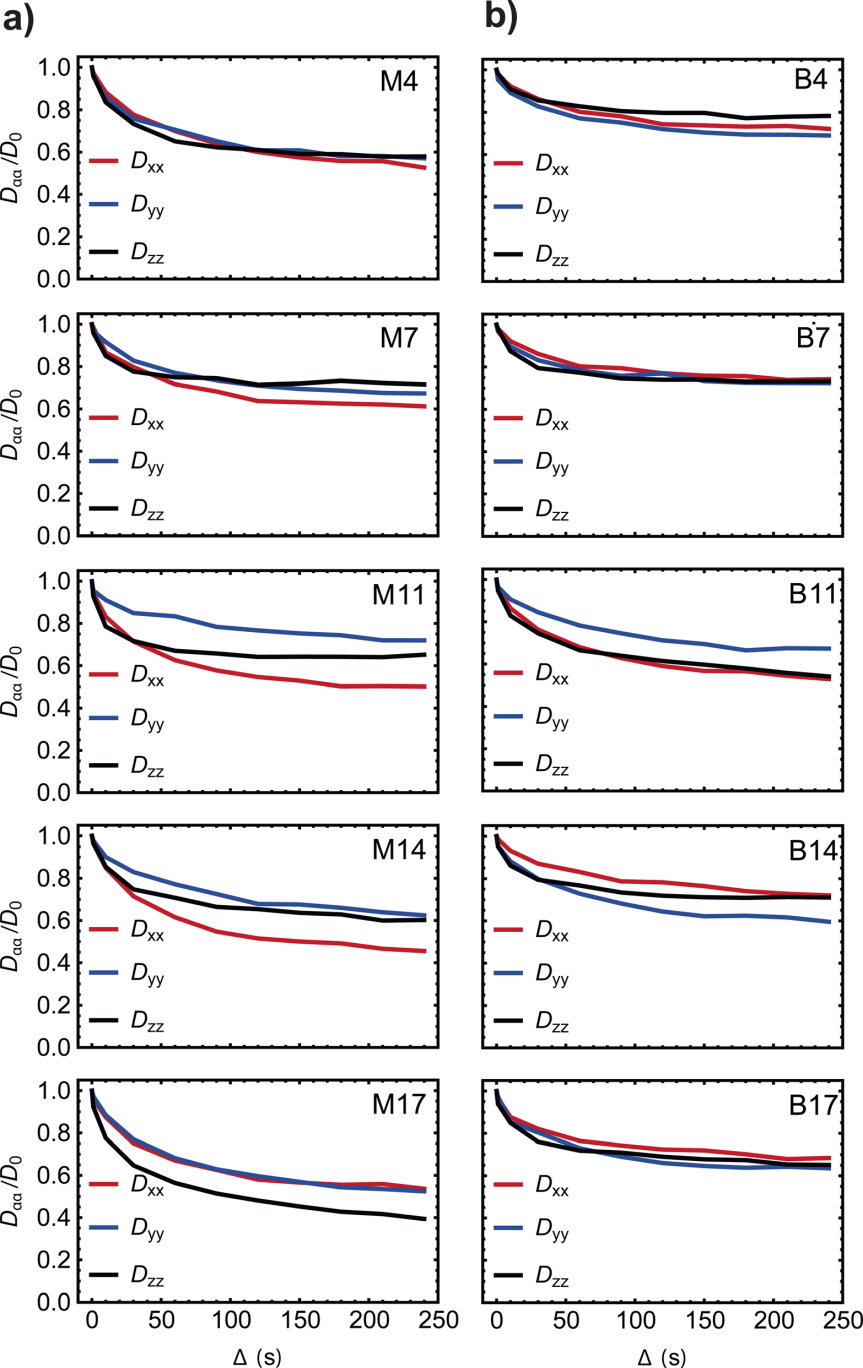
Simulated values of the diffusion coefficients along the three main directions of the laboratory frame (solid lines, normalised to *D*_0_) plotted against the diffusion time *Δ* for samples (a) M4–M17 and (b) B4–B17.

The plots of the simulated diffusion coefficients along the laboratory *x*, *y* and *z* directions for each sample (see Fig. 10) contain some other important information. To understand how to read these data, let us first imagine how cells proliferate on these 3D supports.

It is reasonable to think that at the beginning of the culture, there are very few cells and these are essentially attached to the fibres and there is not much change in the pore space. Diffusion is therefore expected to be similar along the *x*, *y* and *z* directions of the laboratory (here *z* coincides with the cylindrical scaffold main axis, whereas *x* and *y* are the perpendicular directions where the scaffold develops in a circular shape). Indeed diffusion coefficients are similar in all three directions for samples M4 and B4. As the culture time is increased and the cells proliferate and infiltrate the pore space, pores are expected to get smaller but the extent of the infiltration and the coverage of cells within the scaffold is not easily predictable and depends on many factors including the quality of the seeding, scaffold adherence treatment, culture protocol, and so on. Generically speaking one can expect that the pores become more anisotropic after a while, meaning that the diffusion coefficients along the three directions are expected to depart from each other. This is seen in Fig. 10 for sample M7, M11, M14, M17, B7, B11, and B14. Eventually, if the culture time is long enough and the cells infiltrate homogeneously the pore space and close up most of the pores one expects that the diffusion coefficients along the three directions return to be identical to each other to reflect a more isotropic space. This is observed in B17 but not in M17 where probably the culture needed a few more days to infiltrate the pore space more homogeneously. Furthermore, one also expects that the asymptotic value reached at long diffusion times is lower at long diffusion times than at the beginning of the culture since the pore space has now become more tortuous. This is again observed in our simulation (compare B4 to B17).

## Conclusions

In this paper, we have introduced a simulation framework to calculate the diffusion tensor, and some related quantities, in porous media of arbitrary complexity. The algorithm is based on a simple random walk approach where molecules are let to diffuse randomly in the actual porous structure of the medium that has been digitised, with micrometric precision, from μCT images. The framework has been tested on *in silico* structures first, and then on model porous media made up of randomly packed polyethylene beads for which we have experimental diffusion data measured *via* nuclear magnetic resonance techniques. Finally, the validated framework has been applied to study the diffusion of molecules inside the space and time heterogeneous pore structure of a tissue growing on 3D-printed plastic scaffolds, as those used in tissue engineering for regenerative medicine or for 3D models of cancer. Our results have shown that it is possible to correlate the value of tortuosity, a quantity that can be extracted from diffusion tensor calculations, with tissue proliferation. Understanding how the pore structure changes over space and through time in this “live” system is an important piece of information since the delivery of nutrients and removal of waste is crucial for the correct development of the tissue itself and therefore such information can be used to design better scaffolds and/or better culture protocols for tissue engineering.

## Data availability

The simulation routines generated in this paper are available at: https://github.com/Topaz765/DTI-Porous-Media.

## Author contributions

TAAC: software, investigation, formal analysis, visualization, writing—review and editing. YW: cell culture, investigation, visualization, writing—review and editing. TBRR: experiments, visualization, data analysis, writing—review and editing. OLK: experiments, data analysis, writing—review and editing. GP: conceptualization, methodology, formal analysis, visualization, supervision, validation, funding acquisition, writing—original draft.

## Conflicts of interest

There are no conflicts to declare.
